# Targeted sequencing identifies genetic polymorphisms of flavin‐containing monooxygenase genes contributing to susceptibility of nicotine dependence in European American and African American

**DOI:** 10.1002/brb3.651

**Published:** 2017-03-15

**Authors:** Tian‐Xiao Zhang, Nancy L. Saccone, Laura J. Bierut, John P. Rice

**Affiliations:** ^1^ Department of PsychiatryWashington University School of MedicineSt. LouisMOUSA; ^2^ Department of GeneticsWashington University School of MedicineSt. LouisMOUSA

**Keywords:** flavin‐containing monooxygenase, genetic association, rare variants, targeted sequencing

## Abstract

**Background:**

Smoking is a leading cause of preventable death. Early studies based on samples of twins have linked the lifetime smoking practices to genetic predisposition. The flavin‐containing monooxygenase (FMO) protein family consists of a group of enzymes that metabolize drugs and xenobiotics. Both *FMO1* and *FMO3* were potentially susceptible genes for nicotine metabolism process.

**Methods:**

In this study, we investigated the potential of FMO genes to confer risk of nicotine dependence via deep targeted sequencing in 2,820 study subjects comprising 1,583 nicotine dependents and 1,237 controls from European American and African American. Specifically, we focused on the two genomic segments including *FMO1*,*FMO3*, and pseudo gene *FMO6P*, and aimed to investigate the potential association between FMO genes and nicotine dependence. Both common and low‐frequency/rare variants were analyzed using different algorithms. The potential functional significance of SNPs with association signal was investigated with relevant bioinformatics tools.

**Results:**

We identified different clusters of significant common variants in European (with most significant SNP rs6674596, *p *=* *.0004, OR = 0.67, MAF_EA = 0.14, *FMO1*) and African Americans (with the most significant SNP rs6608453, *p *=* *.001, OR = 0.64, MAF_AA = 0.1, *FMO6P*). No significant signals were identified through haplotype‐based analyses. Gene network investigation indicated that both *FMO1* and *FMO3* have a strong relation with a variety of genes belonging to *CYP* gene families (with combined score greater than 0.9). Most of the significant variants identified were SNPs located within intron regions or with unknown functional significance, indicating a need for future work to understand the underlying functional significance of these signals.

**Conclusions:**

Our findings indicated significant association between FMO genes and nicotine dependence. Replications of our findings in other ethnic groups were needed in the future. Most of the significant variants identified were SNPs located within intronic regions or with unknown functional significance, indicating a need for future work to understand the underlying functional significance of these signals.

## 1 Introduction

Smoking is a leading cause of preventable death, causing about 5 million premature deaths worldwide each year, and current trends show that tobacco use will cause more than 8 million deaths annually by 2030 (WHO, 2013). Strong evidence connects cigarette smoking and lung cancer (Biesalski et al., 1998; Doll & Hill, 1954; Doll, Peto, Boreham, & Sutherland, 2004; Hecht, 2012), and according to the data from American cancer society, lung cancer causes the most death each year compared to other cancers (Shafey, Eriksen, Ross, & Mackay, 2009). In addition, cigarette smoking is also the principal environmental risk factor for developing chronic obstructive pulmonary disease, a disease characterized by chronically poor airflow (Kennedy, Chambers, Du, & Dimich‐Ward, 2007; Laniado‐Laborín, 2009; Tønnesen, 2013). Therefore, understanding the underlying biological mechanisms of nicotine dependence will still have huge public health significance in the future.

Early studies based on samples of twins have linked the lifetime smoking practices to genetic predisposition (Carmelli, Swan, Robinette, & Fabsitz, 1992). A meta‐analysis of the data from five studies, each involving more than 1,000 twin pairs, showed an estimated heritability of 60% for the propensity to smoke (Vink, Willemsen, & Boomsma, 2005). The followed linkage and gene association mapping studies have identified several susceptible loci, including genes encoding dopamine transporter/receptors (Huang et al., 2008; Lerman et al., 1999; Sabol et al., 1999), cholinergic receptors (Feng et al., 2004; Hong et al., 2010; Saccone et al., 2006; Thorgeirsson et al., 2010), taste receptor (Mangold, Payne, Ma, Chen, & Li, 2008), serotonin receptor (Gerra et al., 2005; Kremer et al., 2005), and gamma‐aminobutyric acid type B receptor (Beuten et al., 2005), that are associated with nicotine dependence. The breakthrough of microarray technology at the end of 20th century enabled the “unbiased” association mapping analysis in the whole human genome. Genome‐wide association study (GWAS), which scans the whole genome by capturing the information of common SNPs, has been proved informative for nicotine dependence (Bierut et al., 2007; Liu et al., 2009; Thorgeirsson et al., 2008; Uhl et al., 2007), and greatly accelerates the progress of this gene hunting process. Nevertheless, GWAS only focuses on a set of preselected, generally common SNPs, and tends to omit the rare variants and structural variants such as short insertion and deletions (indels). The recent development of “next‐generation” sequencing technology has enabled researchers to investigate these variants which are not covered in GWAS at a relatively lower genotyping cost (Hall, 2007; Quail et al., 2012; Tucker, Marra, & Friedman, 2009). A recent published study focusing on targeted sequencing data of *CHRNA5* has identified several novel rare and low‐frequency coding variants that contributed to nicotine dependence (Olfson et al., 2015).

Three protein families are involved in nicotine pharmacokinetics: liver cytochrome P450 enzymes (CYPs), flavin‐containing monooxygenases (FMOs), and uridine diphosphate glucuronosyltransferase enzymes (Bloom et al., 2013). The FMO protein family consists of a group of enzymes that metabolize drugs and xenobiotics (Krueger & Williams, 2005). Five forms of FMOs are found in human and have been designated *FMO1*–*FMO5* (Krueger & Williams, 2005). Among these FMO genes, part of nicotine inhaled during smoking can be broken down to *N*′‐oxide by FMO 3 (encoded by *FMO3*) (Bloom et al., 2013). Hinrichs et al. (2011) has identified significant association between SNPs of *FMO1* and nicotine dependence. Although a recent study has shown that common polymorphisms in *FMO3* can influence nicotine clearance (Bloom et al., 2013), no study has provided direct evidence of the association between *FMO3* polymorphisms and nicotine dependence. Except for these five forms of FMOs found in human, *FMO6P*, which is a pseudo gene located very near to *FMO3*, is reported to have significant sequence homology with *FMO3* (Hines, Hopp, Franco, Saeian, & Begun, 2002). To sum up, both *FMO1* and *FMO3* were potentially susceptible genes for nicotine metabolism process and further studies were still needed to investigate the underlying variants within these genes that are associated with the susceptibility of nicotine dependence.

In this study, we investigated the potential of FMO genes to confer risk of nicotine dependence via deep targeted sequencing in 2,820 study subjects (1,432 European and 1,388 African Americans) comprising 1,583 nicotine dependents and 1,237 controls. Specifically, we focused on the two genomic segments including *FMO1*,* FMO3* (protein coding genes for FMO 1 and 3), and *FMO6P* (pseudo gene), and aimed to investigate the potential association between FMO genes and nicotine dependence. Via implementing targeted sequencing, we are interested to figure out that whether rare variants contribute to the association signal derived from common variants. In addition, comparisons were made between the association results based on European Americans and African Americans.

## 2 Methods

This research was reviewed and approved by the Institutional Review Board at Washington University in Saint Louis. All the study subjects provided informed consent.

### 2.1 Study subjects and sample ascertainment

Study subjects were recruited from Collaborative Genetics Study of Nicotine Dependence and the Genetic Study of Nicotine Dependence in African Americans (Bierut et al., 2007; Saccone et al., 2006). A total of 2,820 individuals comprising 1,432 European Americans and 1,388 African Americans were examined in our study. We assessed the study subjects’ smoking behavior using Fagerström test for nicotine dependence (FTND; Heatherton, Kozlowski, Frecker, & Fagerström, 1991). The nicotine dependence patients were defined as current smokers with FTND score equal or greater than 4, and controls were defined as having FTND score of 0 or 1 and have smoked at least 100 cigarettes in their lifetime (Table 1).

**Table 1:** Characteristics of study subjects

	Nicotine dependent	Nondependent
Sample, *n*	1,583	1,237
Gender (%)		
Female	901 (59)	805 (65)
Male	682 (41)	432 (35)
Ethnicity (%)		
European American	730 (46)	702 (57)
African American	853 (54)	535 (43)
Age in year, mean (range)	37 (25–45)	36 (25–45)
FTND score, mean (range)	6.34 (4–10)	0.16 (0–1)

FTND, Fagerström test for nicotine dependence.

### 2.2 Targeted sequencing of *FMO1* and *FMO3*

DNA samples were extracted from blood with Puragene. Targeted sequencings on two 100 kb regions of *FMO1* and *FMO3* were performed at the Center for Inherited Disease Research. These genomic regions also contain part of gene *FMO4* and a whole pseudo gene *FMO6P*. The quality control was implemented in samples and variants level, respectively. The mean on‐target coverage was 180× for each sequencing experiment and greater than 96% of on‐target bases had a depth greater than 20×. Full detailed description of genotyping and quality control process can be found in Supplemental Methods.

### 2.3 Statistical analysis

A total of 1,432 European Americans and 1,388 African Americans with targeted sequencing of *FMO1* and *FMO3* were examined. General data analyses were performed by R (R i386 3.2.1; Ripley, 2001). To quantify the potential population stratification, we conducted principal component analysis (PCA) in the combined sample (115,338 markers), as well as separately in the European American sample (154,049 markers) and African American sample (218,399 markers), using a previous collected genome‐wide array dataset containing 950,847 SNPs (Price et al., 2006). Population genetics software STRUCTURE was utilized to conduct PCA (Pritchard, Stephens, & Donnelly, 2000). Sequencing data were annotated by sequencing data annotation software Annovar (Wang, Li, & Hakonarson, 2010). We have implemented a comprehensive approach for the quality control of our targeted sequencing data and detail information and the criteria used can be found in supplemental methods. After variant‐level quality control, 5,105, 2,600, and 3,817 variants located within the two targeted genomic regions (*FMO1*/*FMO3*) were extracted from combined, European American, and African American sample set, respectively. Sixty‐four variants with functional significance (nonsynonymous/stop–gain variants or frameshift indels) were identified (Table S1).

Variants satisfying the following criteria were utilized in variant‐level analysis: (1) variants with MAF > 0.05 and (2) located within targeted gene regions or the linkage disequilibrium (LD) blocks that are (partly) overlapped with the targeted gene regions (detailed definition of blocks is given below). The association analysis was conducted by fitting logistic regression model. The genotypic data were coded in additive model. This analysis was performed in combined, European American, and African American individuals separately (for combined subjects, we tested a union of SNPs sets selected based on European American and African American subjects). Gender and age were included as covariates in all the three analyses. The first two principal components based on the three sample sets were also utilized as covariates accounting for the potential population stratification when fitting the logistic models. To address the multiple comparison problem, we implemented Bonferroni correction. The number of tests was calculated in the following way:

$$N=n_{1}+n_{2},$$

where for each dataset, *n*_1_ stands for the number of LD blocks generated by this dataset and *n*_2_ is the number of variants that do not belong to any LD blocks. In addition to testing associations for single variants, we also conducted haplotype‐based analysis with combination of multiple variants in European American and African American datasets, respectively. LD blocks were constructed using the default algorithm taken from Gabriel et al. (2002). On *D*′, 95% confidence bounds are generated and each comparison is called “strong LD” when the confidence bounds have upper bound ≥0.98 and lower bound ≥0.7, and a block is created if 95% of informative comparisons are “strong LD.” Variant‐level association analysis and LD construction and haplotype analyses were conducted using Plink (Purcell et al., 2007). Bonferroni correction was also applied to haplotype‐based analyses. Six haplotypes were constructed and tested in European Americans (Tables S7), while 20 haplotypes were constructed and tested in African Americans (Tables S7). The *p*‐value thresholds used for European Americans and African Americans were .008 and .0025, respectively.

Variants were then classified into two categories for the gene‐level analysis (mostly rare variants). The two categories are: (1) gene‐region variants set (i.e., the variants located within the gene region) and (2) functional‐region variants (variants located within regions with significant functional significance, including exonic regions, 3′/5′ UTR, smaller comparing to gene‐region set). For these two variants sets, analysis was performed on variants with MAF less than 0.01 and 0.05. Both SKAT and weighted burden test (Lee et al., 2012) were utilized for the gene‐level analysis. Same as the variant‐level analysis, we also conducted this analysis in combined, European American, and African American individuals separately. Gender, age, and first two PCs based on the three sample sets were also included as covariates. LocusZoom was utilized to make regional association plots (Pruim et al., 2010).

### 2.4 Bioinformatics analysis

We examined the targeted SNPs and/or genes using several bioinformatics tools and databases. We utilized the protein–protein interaction database STRING (http://string-db.org/; Szklarczyk et al., 2015) to explore the potential interactions of our targeted genes. The Regulome DB (http://regulomedb.org/; Boyle et al., 2012) was used to predict the potential functional consequences of the identified risk SNPs. This database is a web‐based bioinformatics tool integrated with multiple types of data (including ChIP‐seq, DNase‐seq, eQTLs, etc.) from the Encyclopedia of DNA Elements (ENCODE) project (Xie et al., 2013).

## 3 Results

### 3.1 Variant‐wise association of FMO genes and nicotine dependence

**Figure 1:**
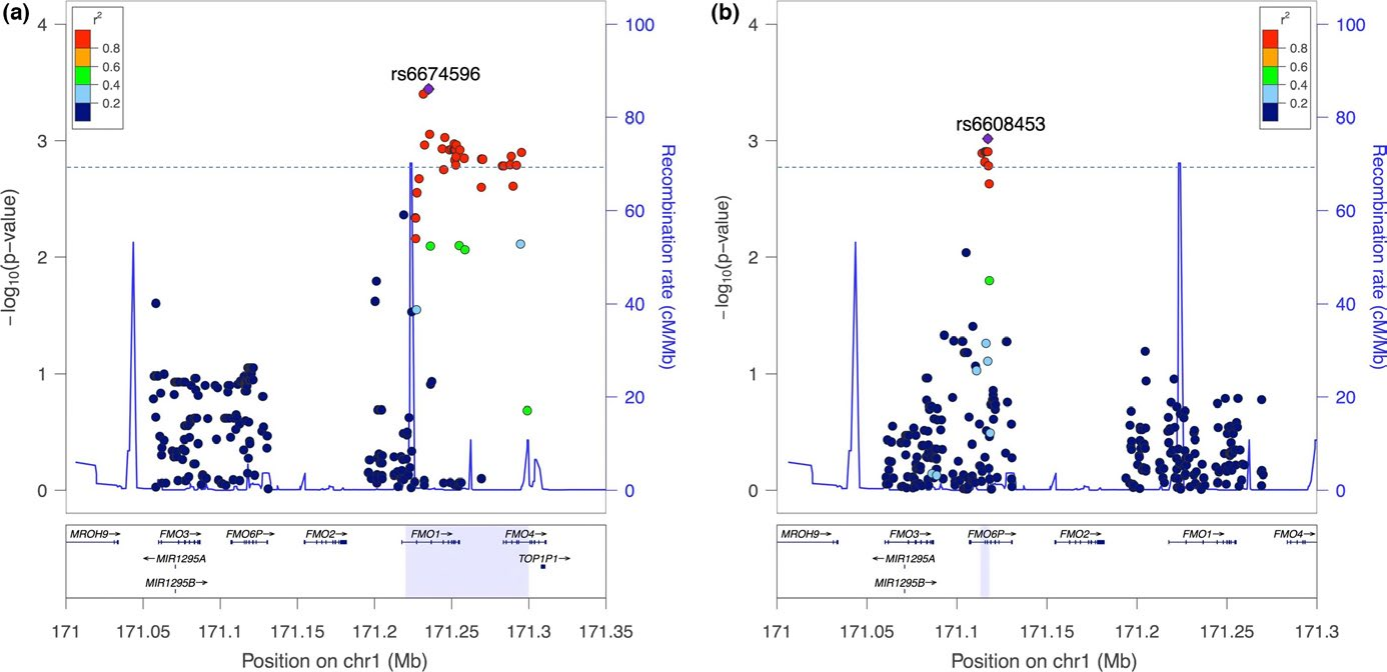
Regional association plots of *FMO1–FMO3–FMO6P* genomic region based on (a) European Americans and (b) African Americans. The blue dash lines are the −log_10_ (*p*‐value) threshold used in our study(1.25 × 10^−3^)

Variants (270, 326, and 368) were selected for variant‐wise association analysis in European American, African American, and combined sample set, respectively. LD blocks were constructed in European American and African American sample sets (Tables S2 and S3). Based on these LD blocks patterns, the significant thresholds for variant‐wise analysis were 3.6 × 10^−3^ and 1.25 × 10^−3^ in European American and African American sample set, respectively. We chose the most conservative one as our *p*‐value threshold in the analysis (1.25 × 10^−3^). Multiple different significant variants were identified in European American and African American datasets (Table 2, Figure 1). A cluster of significant variants were identified in European American individuals (with most significant SNP rs6674596, *p *=* *.0004, OR = 0.67, *FMO1*). In African American individuals, we identified several clustered significant variants (with the most significant SNP rs6608453, *p *=* *.001) in pseudo gene *FMO6P*. The full results of variant‐wise association analysis were summarized in Tables S4–S6.

**Table 2:** Significant signals in variant‐wise association analysis

CHR	VAR	GENE	POS	A1	OR_EA	*p*_EA	MAF_EA	OR_AA	*p*_AA	MAF_AA
1	rs11812044	*FMO6P*	171115567	A	1.16	.1232	0.19	0.65	**.0012**	0.10
1	rs17565793	*FMO6P*	171116267	C	1.16	.1232	0.19	0.65	**.0012**	0.10
1	rs17623477	*FMO6P*	171116304	C	1.16	.1232	0.19	0.65	**.0012**	0.10
1	rs7051747	*FMO6P*	171116550	G	1.16	.1232	0.19	0.65	**.0012**	0.10
1	rs7066454	*FMO6P*	171116603	T	1.16	.1232	0.19	0.65	**.0012**	0.10
1	rs7063044	*FMO6P*	171116760	T	1.16	.1232	0.19	0.65	**.0012**	0.10
1	rs6608453	*FMO6P*	171117140	T	1.17	.1109	0.19	0.64	**.0010**	0.10
1	rs6608454	*FMO6P*	171117170	C	1.16	.1232	0.19	0.65	**.0012**	0.10
1	rs12726624	*FMO1*	171231630	G	0.67	**.0004**	0.13	1.03	.7385	0.47
1	rs17581251	*FMO1*	171232446	T	0.67	**.0011**	0.12	0.98	.8685	0.08
1	rs28360379_indel	*FMO1*	171234851	A	0.68	**.0006**	0.13	0.99	.8698	0.43
1	rs6674596	*FMO1*	171235088	T	0.67	**.0004**	0.14	1.01	.9325	0.46
1	rs13376631	*FMO1*	171235742	G	0.69	**.0009**	0.13	1.00	.9831	0.43
1	rs12094878	*FMO1*	171243863	C	0.69	**.0012**	0.14	1.04	.6315	0.47
1	rs12062692	*FMO1*	171245579	G	0.69	**.0009**	0.14	0.97	.7002	0.48
1	rs7539057	*FMO1*	171248614	A	0.70	**.0012**	0.14	0.95	.4840	0.42
1	rs742350	*FMO1*	171250044	T	0.69	**.0012**	0.14	1.03	.6911	0.46
1	rs12091482	*FMO1*	171251509	T	0.70	**.0012**	0.14	0.94	.4723	0.42
1	rs10399952	*FMO1*	171251663	G	0.69	**.0011**	0.14	0.95	.5037	0.42
1	rs10399602	*FMO1*	171251876	C	0.70	**.0012**	0.14	0.95	.4840	0.42
1	rs7519999	*FMO1*	171251958	G	0.70	**.0012**	0.14	0.95	.4840	0.42
1	rs1126692	*FMO1*	171252287	G	0.70	**.0012**	0.14	0.93	.3914	0.46
1	rs12092985	*FMO1*	171252537	A	0.70	**.0012**	0.14	0.95	.4840	0.42
1	rs10912714	*FMO1*	171253037	G	0.69	**.0011**	0.14	0.95	.4840	0.42
1	rs12059179	*FMO1*	171255346	T	0.70	**.0012**	0.14	0.91	.2620	0.39

Significant findings were given in bold.

### 3.2 Haplotype‐based and gene‐wise association of FMO genes and nicotine dependence

We performed haplotype‐based analyses in European American and African American dataset separately. The *p*‐value thresholds were decided by Bonferroni correction and thus were different for each dataset. We utilized .008 (.05/6) and .0025 (.05/20) as *p*‐value threshold for European American and African American dataset, respectively. No significant signals were identified through haplotype‐based analyses. The full results of haplotype‐based analyses were summarized in Tables S7 and S8. Gene‐wise association analyses mainly focused on rare and/or low‐frequency variants in our dataset. Although we have tried multiple analytical schema (combination of different MAF threshold and region definitions), no significant association signals were found in this analysis (Table S9).

### 3.3 Bioinformatics analysis

Proteins that show evidence for interaction with proteins encoded by *FMO1*,* FMO3*, and *FMO6P* were extracted from STRING (Figs S1–S3, Tables S10–S12). STRING provided a combined score for each protein pair as an evaluation of the relation between the two proteins, and this score was scaled from 0 to 1. Higher score indicates stronger relation between the two proteins. Both *FMO1* and *FMO3* have a strong relation with a variety of genes belonging to CYP gene families (with combined score greater than 0.9). *FMO6P*, however, as a pseudo gene, only showed limited evidence to be related with *PRSS16*, which encodes a serine protease expressed exclusively in the thymus (with combined score of 0.409).

We explored the top significant variants (rs6674596 and rs608453) in Regulome DB to investigate their potential biological significance. Regulome DB has its own scoring system to measure the biological significance of a variant. The range of the scores is from 1 to 6, and the smaller the score is, the more evidence that indicate this variant has biological significance. rs6674596 has a Regulome DB score of 5, and is located within a DNase hypersensitive area of assayed in multiple cell types. In addition, this variant is also located at a sequence motif region (HNF1). rs608453 has a Regulome DB score of 6. It is also located in a sequence motif (Cdx). No expression quantitative trait loci (eQTL) or transcription factor binding related evidence was shown for neither variants.

## 4 Discussion

As part of a large‐scale targeted sequencing study focusing on nicotine‐dependent/nondependent smokers, our aim was to test the hypothesis that genetic polymorphisms of FMO genes contribute to the risk of nicotine dependence. The underlying rationale of this study is based on the fact that FMO genes are key genes of the nicotine metabolism pathway (Bloom et al., 2013). *FMO1* may play a role in nicotine metabolism and contributed to the nicotine level in brain organ (Mackillop, Obasi, Amlung, McGeary, & Knopik, 2010), and *FMO3* encodes FMO 3, which can metabolize a small percentage of nicotine into nicotine *N*′‐oxide (Bloom et al., 2013). We studied both rare and common variants in *FMO1*,* FMO3*, and *FMO6P* through large‐scale targeted sequencing.

A number of common variants in *FMO1* were identified to be significantly associated with nicotine dependence, and we noted that there was an ethnic‐specific pattern. We identified a cluster of significant variants in *FMO1* in the European Americans. The most significant variant was rs6674596 (*p *=* *.0004, OR* *=* *0.67, MAF_EA* *=* *0.135,). However, this significant result was not replicated in our African American dataset (*p *=* *.9325, OR* *=* *1.01, MAF_AA* *=* *0.463). The association signals for *FMO1* have been reported by Hinrichs et al. (2011). Several significant SNPs reported in that study, including rs742350 and rs1126692, were also identified to be significant in our study. Considering both studies utilized COGEN samples, our results on the common SNPs basically replicated Hinrichs’ results. In addition to the significant findings in European American sample set, we also identified a set of significant variants located on gene *FMO6P* from the African American dataset (with the most significant SNP rs6608453, *p *=* *.001, MAF_AA* *=* *0.097). Just like significant variants were only identified in European Americans, this significant signal of *FMO6P* was only identified in African Americans, but failed to be replicated in European Americans (*p *=* *.1109, OR* *=* *1.17, MAF_EA* *=* *0.192). No significant SNPs were identified from the combined sample set; although in the combined samples set, the sample size almost doubled with a correspondent increment in statistical power. The reasons behind this ethnic‐specific pattern might be complex, and the most plausible one is differences in the regional LD structure between the two racial/ethnic groups. This difference might mean the surrogate SNPs miss the signal created by the real underlying susceptible variants in a specific set of samples.

One major advantage of our targeted sequencing study is that we can examine every possible DNA variations in our targeted regions and conducted association analysis thoroughly. However, in this study, we did not detect significant association between the rare variants and nicotine dependence, although we systematically tried many combinations of statistical methods, MAFs, region definitions, and sample sets. The most significant rare variant set was identified for gene *FMO1* with region definition of “gene region” and MAF < 0.01 in African Americans (*p *=* *.0636). The lack of significant findings for rare variants suggests that the significant associations for common SNPs are not simply surrogates for rare variant associations (synthetic associations; Dickson, Wang, Krantz, Hakonarson, & Goldstein, 2010).

We found it interesting to examine the functional significance of the significant common SNPs we identified. All of the significant common SNPs are located either in introns or outside the gene. Therefore, if these significant common SNPs alter function, it is not by changing protein structure. The most significant SNP in *FMO1*, rs6674596, is located within a DNase hypersensitive area of assayed in multiple cell types, and most of the regulatory regions and some promoter regions tend to be DNase sensitive. This suggests that this SNP might have an effect on the expression of gene *FMO1*. Nevertheless, without further evidence from biological experiments, it is still too early to explain this association signal.

*FMO6P* is a pseudo gene which means that this gene cannot be properly expressed as a protein, and it is probably because it is unable to produce a full‐length transcript (Hines et al., 2002). *FMO6P* is reported to have significant sequence homology with *FMO3* (Hines et al., 2002). Previous studies have set up direct links of SNPs in *FMO6P* with chronic allograft dysfunction (Israni et al., 2013) and pharmacokinetic characteristics of sulindac sulfide in premature labor (Park et al., 2014). One interesting note for these previous studies is that the significant findings of *FMO6P* are always accompanied with significant findings from *FMO3*, and at least in one study (Israni et al., 2013), the significant SNP of *FMO6P* is in complete LD with significant SNP of *FMO3*. This suggests that the significant hit in *FMO6P* might be a surrogate for some true underlying signal in *FMO3*. However, in our study, although some SNPs of *FMO3* are indeed in complete LD with the significant SNPs of *FMO6P*, the whole significant SNP cluster is located in the *FMO6P* region (Figure 1). On the other hand, if the signal we identified in *FMO6P* is not the surrogate for effects of SNPs in *FMO3*, but has an independent effect on nicotine dependence, then further research will be needed to clarify the underlying function of *FMO6P*.

A major strength of our study is that, unlike most of the common SNP‐based association studies, we implemented a targeted sequencing technology for genotyping of our study subjects. This enables us to consider both common and rare variants within the three gene regions. Additionally, this study design enabled us to analyze both SNVs and indels which are often omitted in SNP‐based study designs. However, there are also some limitations to this study that need to be noted. First, we lack replication for our significant findings. The design using two racial/ethnic groups in our study enabled us to use as the two datasets as replication set for each other. However, significant findings in the European American dataset were not confirmed in the African American dataset. In addition, our sample size limited the statistical power to detect potential modest effects of SNPs. This is a common challenge, especially when using study designs, such as targeted sequencing, which generate genotype data at many variants, leading to multiple comparisons and corresponding stringent significance requirements. Future work to address this challenge would be to combine multiple sequenced datasets using meta‐analysis, such approaches have been productive for GWAS of complex traits and have yet to be fully leveraged for sequencing studies and rare variant analyses.

In summary, we tested the genetic effects of three FMOs genes (*FMO1*,* FMO3*, and *FMO6P*) on nicotine dependence by performing targeted sequencing on 2,852 nicotine‐dependent and nondependent smokers. We performed both variant‐level and gene/region‐level analyses to examine the genetic association of rare, low‐frequency, and common variants within these region and nicotine dependence, and both SNVs and indels. We identified significant association signals for gene *FMO1* and *FMO6P*. Replications of our finds in other ethnic groups were needed in the future. Most of the significant variants identified were SNPs located within intron regions or with unknown functional significance, indicating a need for future work to understand the underlying functional significance of these signals.

## Authors’ Contributions

TXZ, LJB, and JPR were responsible for the study concept and design. TXZ performed the statistical analysis. NLS, LJB, and JPR assisted with data analysis and interpretation of findings. TXZ drafted the manuscript. NLS, LJB, and JPR provided critical revision of the manuscript for important intellectual content. All authors critically reviewed content and approved final version for publication.

## Conflict of Interest

LJB, JPR, and the spouse of NLS are listed as inventors on Issued U.S. Patent 8,080,371, “Markers for Addiction” covering the use of certain SNPs in determining the diagnosis, prognosis, and treatment of addiction.
