# Supplementary material for: Targeted sequencing identifies genetic polymorphisms of flavin‐containing monooxygenase genes contributing to susceptibility of nicotine dependence in European American and African American
Source: Brain Behav. 2017 Mar 15;7(4):e00651. doi: 10.1002/brb3.651 (PMC5390834; doi:10.1002/brb3.651)
Supplement: Supplementary file 1 [file BRB3-7-e00651-s001.docx]

**Supplemental Methods**

*Targeted sequencing of FMO1 and FMO3*

Samples were placed on 96-well plates for library preparation and capture. The plates were stratified by case/control status, recruitment site, race, and sex, with samples distributed according to the incidence of each in the project. Captures of 50 kilobases on either sided of the genes were done with the Agilent SureSelect XT Custom ELID 0414301. A range of 500ng to 1ug of genomic DNA was sheared using the Covaris E-210 instrument using modified parameters for shearing (DutyCycle=10%, Intensity=4, Cycles per Burst=200, time=80sec). Samples were clustered putting between 48-96 per flow cell lane using the Illumina cBot Paired End Cluster Generation Kit with v3 HiSeq 2000 flow cell (Illumina). One hundred bp paired-end sequencing was performed on the HiSeq2000 with TruSeq SBS v3 chemistry (Illumina). Intensity analysis and base calling were performed through the Illumina Real Time Analysis (RTA) software (version 1.17.20). 3 Fastq files were aligned with BWA [1] version 0.5.10-tpx to the 1000 genomes phase 2 (GRCh37) [2]. Genome Analysis Toolkit (GATK) version 2.3-9-ge5ebf34. Unified Genotyper (GATK) was used for multi-sample calling of SNVs and indels using Reduced BAMs (GATK). Unified Genotyper was used for multi-sample calling and VQSR for variant filtering. All samples had 96-SNP barcode genotyping for sample identity tracking and concordance checking.

*Quality Control Measures*

Data quality was systematically evaluated using a robust alignment and variant calling workflow implemented by CIDR (<http://www.cidr.jhmi.edu/index.html>). Over 100 quality control metrics were evaluated in real time to quickly identify potential errors and implement fixes throughout the sequencing process. Briefly, sample quality controls were conducted based on batch effects, discordance with array data ,alternate callsets, relatedness and some research specific criteria. Strategies used for variant quality control includes VQSR, duplicate sample discordance, Mendelian errors, Hardy-Weinberg equilibrium (HWE), sequence context, locus report by gene and genotype missing rate. All variants passed the Variant Quality Score Recalibration with a mean quality score of 99, mean depth of 122 with no missing calls, no Mendelian errors and zero discordances between duplicate samples. Importantly, all the rare variants were then manually evaluated by the Quality Assurance/Quality Control analysis team.

Reference:

1. Abecasis GR, Auton A, Brooks LD, DePristo MA, Durbin RM, Handsaker RE, et al (2012). An integrated map of genetic variation from 1,092 human genomes. Nature 491(7422): 56-65.
2. Li H, Durbin R (2010). Fast and accurate long-read alignment with Burrows-Wheeler transform. Bioinformatics (Oxford, England) 26(5): 589-595

**Supplemental Tables Titles**

**Supplementable Tables S1.** Information of DNA variations with functional significance.

**Supplementable Tables S2.** Information of LD blocks constructed based on common SNPs in European American subjects.

**Supplementable Tables S3.** Information of LD blocks constructed based on common SNPs in African American subjects.

**Supplementable Tables S4.** Full results of variant-wise association analysis in European American subjects.

**Supplementable Tables S5.** Full results of variant-wise association analysis in African American subjects.

**Supplementable Tables S6.** Full results of variant-wise association analysis in combined subjects.

**Supplementable Tables S7.** Full results of haplotype based association analysis in European American subjects.

**Supplementable Tables S8.** Full results of haplotype based association analysis in African American subjects.

**Supplementable Tables S9.** Full results of gene-wise association analysis.

**Supplementable Tables S10.** Full results of protein-protein interaction analysis using STRING for FMO1.

**Supplementable Tables S11.** Full results of protein-protein interaction analysis using STRING for FMO3.

**Supplementable Tables S12.** Full results of protein-protein interaction analysis using STRING for FMO6P.

**Supplemental figures**

**
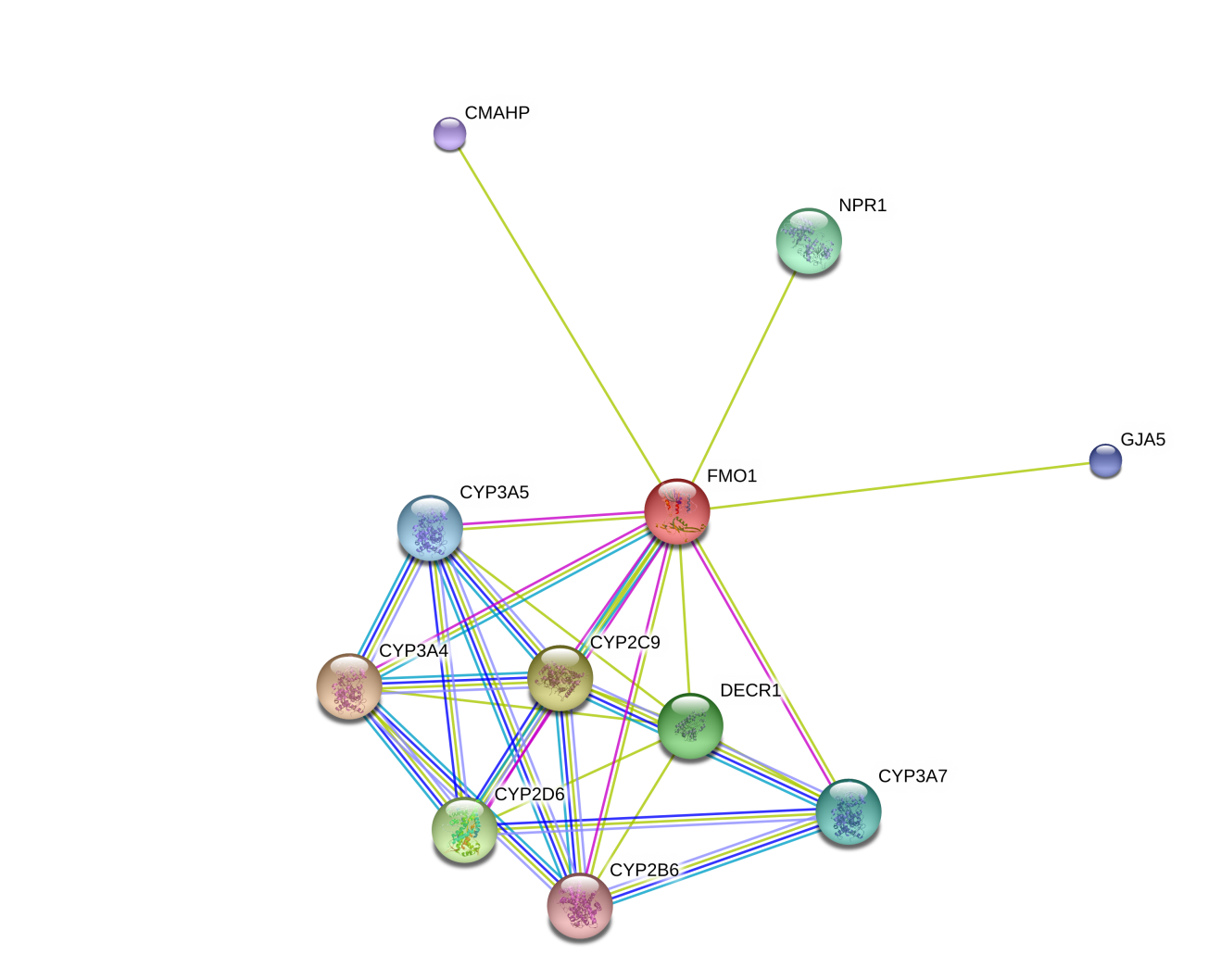
**

**Supplemental Figure S1. Protein-protein interaction network of *FMO1.***


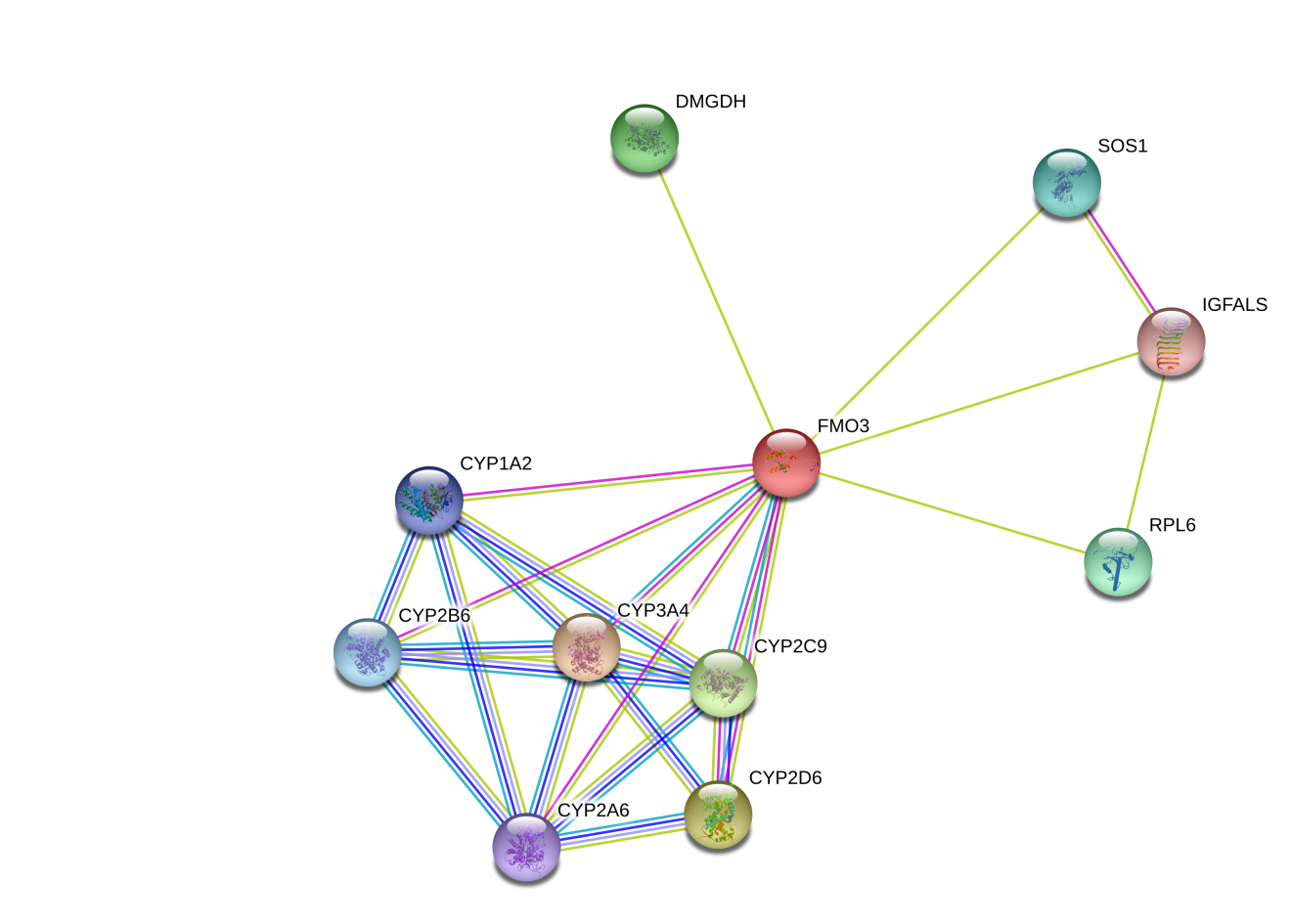


**Supplemental Figure S2. Protein-protein interaction network of *FMO3.***


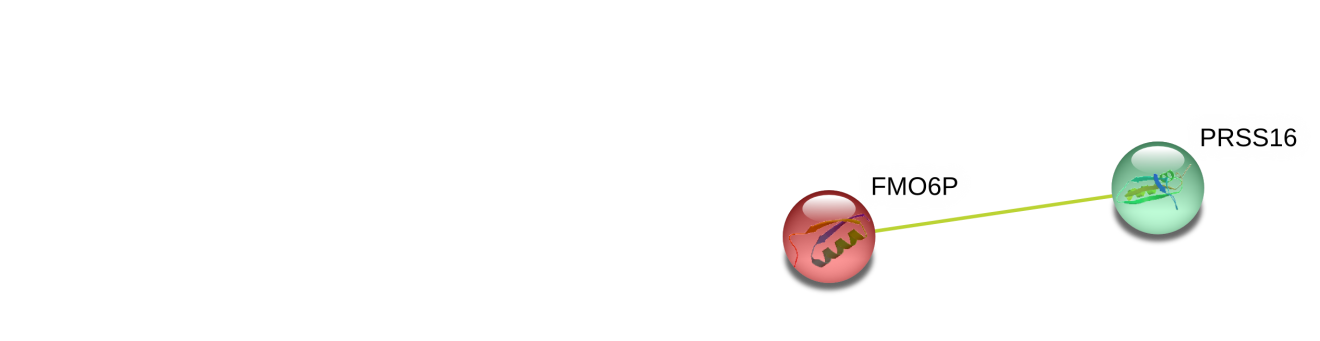


**Supplemental Figure S2. Protein-protein interaction network of *FMO6P.***
